# First-pass contrast bolus hemodynamics contain information on right ventricular function, remodeling, and lung resistance in pulmonary arterial hypertension patients

**DOI:** 10.1186/1532-429X-13-S1-P324

**Published:** 2011-02-02

**Authors:** Jan Skrok, Monda L Shehata, Thomas Goldstein, Jie Zheng, Reda E Girgis, James O Mudd, Joao AC Lima, David A Bluemke, Paul M Hassoun, Jens Vogel-Claussen

**Affiliations:** 1 Johns Hopkins University School of Medicine, Baltimore, MD, USA; 2 Stanford University, Stanford, CA, USA; 3 Washington University School of Medicine, St. Louis, MO, USA; 4 National Institutes of Health, Bethesda, MD, USA

## Background

In pulmonary arterial hypertension (PAH), increased vascular resistance causes functional and structural changes in the right ventricle (RV), ultimately leading to right heart failure and death. Predictors of patient survival include RV cardiac index (RVCI) and pulmonary vascular resistance (PVR). First-pass contrast bolus hemodynamics, such as cardiopulmonary transit time (PTT), full-width-half-maximum (FWHM), and time-to-peak, have been associated with left ventricular (LV) function; however, their relation to RV function and pulmonary hemodynamics as well as their significance in PAH have not been investigated.

## Purpose

To evaluate first-pass contrast bolus hemodynamic parameters in relation to biventricular function and pulmonary hemodynamics in patients undergoing right heart catheterization (RHC) for known or suspected PAH.

## Methods

43 patients (36 females, 58.7 years) underwent RHC and 3T cardiac MRI on the same day. 32 were confirmed to have PAH (mPAP 40 [29-49] mmHg), 11 did not have PAH (mPAP 17 [15-20] mmHg). 18 age- and gender-matched healthy volunteers were included.

**Figure 1:**
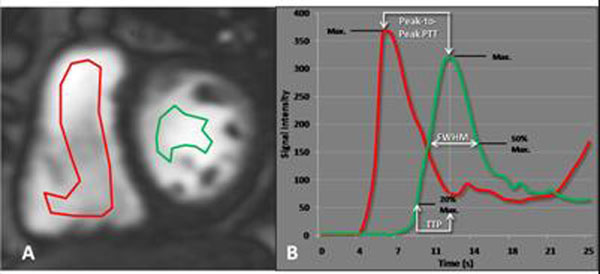
ROI Placement and Time-Intensity Curves. Short-axis saturation-recovery GRE image (A) demonstrates regions of interest placed in the right (red) and left (green) ventricular cavities. Time-intensity curves (B) illustrate the passage of the contrast bolus through the regions of interest and signify the calculated parameters; peak-to-peak cardiopulmonary transit time, full-width-half-maximum (FWHM), and time-to-peak (TTP)

For evaluation of bolus hemodynamics, a 1:10 diluted contrast bolus (0.0025ml/min/kg) of gadopentetate dimeglumine was administered intravenously, and a short-axis saturation-recovery GRE slice was acquired in the proximal third of both ventricles over 40 heartbeats with one image per beat. For analysis, two regions of interest were drawn in the right and left ventricular cavities, and time-intensity curves were generated. From these curves, PTT, FWHM, and time-to-peak were calculated (Figure 1).

## Results

Right-to-left-ventricular PTT, LV FWHM, and LV time-to-peak for PAH patients (8.2s, 8.2s, 4.8s) were significantly longer than for non-PAH (6.5s, p=0.006; 5.0s, p=0.01; 3.6s, p=0.01) and control subjects (6.4s, p=0.0003; 5.2s, p=0.0004; 3.2s, p<0.0001) (Table 1). There were significant correlations of all three parameters with pulmonary hemodynamics and biventricular function and structure (Table 2). In linear regression analysis, including PVRI, ventricular mass index (VMI), and CI as covariates, PTT was predicted by RVCI and VMI, while FWHM and time-to-peak were predicted by PVRI. In receiver operator characteristics (ROC) analysis for transit time to distinguish between PAH patients with and without right heart failure (RVCI <2.2L/min/m^2^) the area under the ROC curve was 0.82 with a sensitivity of 100% and specificity of 63.6% for a threshold of 8.1s.

**Table 1:** Transit times and contrast bolus dispersion

	PAH (n=32)	Non-PAH (n=11)	Controls (n=18)	P
Peak Transit Time (s)	8.2^††‡‡^ [6.9-9.9]	6.5^††^ [5.6-7.0]	6.4^‡‡^ [5.7-7.1]	0.0003*
FWHM LV (s)	8.2^††‡‡^ [5.7-11.4]	5.0^††^ [4.0-7.3]	5.2^‡‡^ [4.1-6.1]	0.0006*
Time-to-Peak LV (s)	4.8^††‡‡^ [3.9-6.5]	3.6^††^ [2.7-4.0]	3.2^‡‡^ [2.8-3.8]	0.0001*

Peak-to-peak cardiopulmonary transit time, left ventricular full-width-half-maximum (FWHM) and time-to-peak measurements were significantly longer for PAH patients than for non-PAH patients and healthy controls. Mann-Whitney *U* test was used to comparison of all three groups: *p<0.01. Wilcoxon rank-sum text was used for individual group comparisons: PAH vs non-PAH: ^†^ p<0.05, ^††^p<0.01; PAH vs. controls: ^‡^p<0.05, ^‡‡^ p<0.01.

**Table 2:** Correlations of Transit Time and Dispersion Coefficients with Biventricular Function and Pulmonary Hemodynamics

	Peak-to-Peak Transit Time		LV FWHM		LV Time-to-Peak	
	**r**	**p**	**r**	**p**	**r**	**p**

Age	0.23	0.13	0.08	0.64	0.04	0.80
6MWD	-0.35	0.03*	-0.23	0.16	-0.32	0.05*
**RHC Parameters**						
Mean RAP	0.20	0.20	0.19	0.24	0.22	0.16
Mean PAP	0.55	0.0001*	0.50	0.0008*	0.47	0.002*
Systolic PAP	0.57	<0.0001*	0.50	0.0008*	0.45	0.003*
PCWP	0.24	0.13	0.06	0.70	0.21	0.18
PVRI	0.64	<0.0001*	0.56	0.0001*	0.54	0.0002*
RHC RV Cardiac Index	-0/48	0.001*	-0.37	0.02*	-0.43	0.004*
RHC RV Stroke Volume Index	-0.55	0.0001*	-0.54	0.0002*	-0.52	0.0004*
LV Stroke Work Index	-0.48	0.001*	-0.48	0.002*	-0.44	0.003*
RV Stroke Work Index	0.30	0.049*	0.23	0.16	0.21	0.19
**Cardiac MRI Parameters**						
LV ED Volume/BSA	-0.42	0.006*	-0.40	0.009*	-0.29	0.06
LV ES Volume/BSA	-0.23	0.14	-0.14	0.38	0.04	0.004*
LV Stroke Volume Index	-0.47	0.001*	-0.50	0.0009*	-0.44	0.004*
LV Cardiac Index	-0.58	<0.0001*	-0.45	0.003*	-0.38	0.01*
LV EF	-0.16	0.32	-0.31	0.049*	-0.40	0.008*
LV Mass/BSA	-0.10	0.53	-0.29	0.07	-0.09	0.59
RV ED Volume/BSA	0.22	0.16	0.09	0.58	0.15	0.35
RV ES Volume/BSA	0.37	0.01*	0.30	0.05	0.33	0.03*
RV Stroke Volume Index	-0.48	0.001*	-0.52	0.0006*	-0.46	0.002*
RV Cardiac Index	-0.59	<0.0001*	-0.45	0.003*	-0.42	0.006*
RV EF	-0.54	0.0002*	-0.50	0.0008*	-0.52	0.0004*
RV Mass/BSA	0.47	0.001*	0.31	0.045*	0.30	0.05
Total Biventricular Mass/BSA	0.28	0.06	0.11	0.51	0.19	0.24

## Conclusions

While right-to-left-ventricular PTT in PAH patients is mainly predicted by right ventricular cardiac function and biventricular remodeling, time-to-peak and FWHM are associated with pulmonary vascular resistance. Their predictive value regarding patient prognosis warrants further investigation.

